# Iron supplementation should be given in breath-holding spells regardless of anemia

**DOI:** 10.3906/sag-1805-92

**Published:** 2019-02-11

**Authors:** Gürkan GÜRBÜZ, Peren PERK, Turgay ÇOKYAMAN, Özge Berfu GÜRBÜZ

**Affiliations:** 1 Department of Pediatric Neurology, Cengiz Gökçek Children’s Hospital, Gaziantep Turkey2-; 2 Department of Family Medicine, Ministry of Public Health, Gaziantep Turkey

**Keywords:** Breath-holding spell, iron deficiency, electroencephalography

## Abstract

**Background/aim:**

The purpose of this retrospective study was to determine the effectiveness of oral iron therapy in breath-holding spells and evaluation of electrocardiographical changes.

**Materials and methods:**

Three hundred twelve children aged 1–48 months and diagnosed with breath-holding spells between January 2017 and April 2018 were included. Patients’ laboratory findings were compared with 100 patients who had one simple febrile seizure.

**Results:**

Cyanotic breath-holding spells were diagnosed in 85.3% (n = 266) of patients, pallid spells in 5.1% (n = 16), and mixed-type spells in 9.6% (n = 30). Sleep electroencephalograms were applied for all patients, 98.2% (n = 306) of which were normal, while slow background rhythm was determined in 1.2% (n = 4). Epileptic activity was observed in only 2 patients (0.6%). The mean hemoglobin (Hb) value in the breath-holding spell group was 10.1 mg/dL. Patients’ mean corpuscular volume (MCV) was 73 fL. Patients’ Hb and MCV values were statistically significantly lower than those of the control group (P < 0.001). The difference between spell burden was not statistically significant (P = 0.691).  Spell burden decreased equally in both groups.

**Conclusion:**

Oral iron therapy can be administered in breath-holding seizures irrespective of whether or not the patient is anemic.

## 1. Introduction

Breath-holding spells are a nonepileptic paroxysmal phenomenon frequently seen in childhood. They were first reported as ‘voluntary breath holding’ in 1737 by Nicholas Culpepper and were described as “A disease ... in children from anger or grief, when the spirits are much stirred and run from the heart to the diaphragms forcibly and hinder or stop the breath ... but when the passion ceaseth” (1). The reported prevalence in European countries is 3.6%–4.6% (2,3). There are three types: cyanotic, pallid, and mixed type. Pallor resulting from bradycardia and asystole developing secondary to increased vagal tone is seen in pallid seizures. While no change in heart beat occurs in cyanotic breath-holding seizures, a blue color is observed around the mouth and in the face associated with prolonged expiration and a decrease in oxygen saturation. The majority of spells conclude with a deep inspiration, although they may sometimes progress with myoclonic jerks or generalized tonic-clonic seizures (4). Seizures are frequently seen between 6 months and 4 years. Spells occur in association with an emotional or physical stimulus and do not occur during sleep. A good history and physical examination are important in differentiating breath-holding seizures from epileptic seizures and other causes of syncope. Prognosis is benign, and attacks decline with maturation of the autonomic nervous system. Loss of consciousness and seizure-like tonic posture may develop not only in pallid spells but also in prolonged cyanotics. An attack being observed by an experienced specialist is diagnostic. Electroencephalography (EEG) is performed to exclude tonic seizures capable of causing similar findings, and electrocardiography (ECG) is applied to exclude familial cardiac arrhythmias in most centers. Neurological examination is generally normal, while iron deficiency anemia is often present in laboratory tests. 

Iron deficiency affects billions of people worldwide and consists of a three-step process that affects the entire body. Iron stocks are reduced in the first phase and then iron-deficient erythropoiesis follows, ultimately resulting in iron deficiency anemia, which is characterized by decreased hemoglobin (Hb), red blood cell count, and mean corpuscular volume (MCV) (5). Iron deficiency anemia is a nutritional disorder that impairs children’s growth, development, and immune system and leads to pallor and fatigue. 

In childhood, due to high growth rates, iron requirements are increased. Inadequate consumption of iron-containing foods or excessive intake of cow or goat milk, especially in the first 6 months of life, increases the risk of iron deficiency (6).

Iron is essential not only for erythropoiesis but also for cerebral neurotransmission. That may be the reason why febrile seizures and breath-holding spells are much more common in individuals who have iron deficiency (7). For this reason, iron stocks should be evaluated and oral iron therapy should be given in breath-holding spells, even if the patient does not have anemia.

Patients with breath-holding spells have been observed to benefit from oral iron therapy. Piracetam is another option in patients who fail to benefit from iron therapy. Basic principles of treatment include explaining to families that breath-holding spells are a treatable disease and have a good course, and emphasizing the behavior required at the time of attacks.

The purpose of this retrospective study was to determine the effectiveness of oral iron therapy in breath-holding spells and evaluate electrocardiographical changes.

## 2. Materials and methods

In this retrospective study, children aged 1–48 months who administered to our hospital’s pediatric neurology outpatient clinic were included. A total of 350 patients having breath-holding spells were diagnosed out of 25,740 between January 2017 and April 2018. Our patients’ histories and examination findings, laboratory tests, and EEG and ECG results were obtained from patient records. Patients with known cardiac, hematological, or neurological problems and those failing to attend follow-up appointments or who could not be contacted by telephone were excluded.

The spells were classified into cyanotic, pallid, or mixed types according to the skin color change during episodes. Severity of spells was divided into two groups of simple and complicated. Both types start with an emotional or painful experience; the simple type ends with a deep breath and cry while complicated spells generally end with loss of consciousness and seizure-like tonic posture.

All patients were evaluated in terms of age, sex, age at onset of attacks, attack frequency, type of breath-holding spell, delivery history, developmental milestones, laboratory values, EEG, ECG, and presence of epileptic seizures, febrile seizures, or breath-holding spells in first-degree relatives. 

Laboratory values of 100 age- and sex-matched patients under monitoring at the same center and undergoing febrile convulsions were randomly included as the control group whether they had anemia or not. Blood was not collected again from these patients, and their laboratory results were also investigated retrospectively.

Hb, serum iron, serum ferritin, and total iron-binding capacity were examined in basal laboratory tests performed at the time of initial presentation in order to examine the relationship between iron deficiency anemia and breath-holding spells. 

Patients with HB, hematocrit, and MCV levels lower than the limits of age and ferritin values less than 15 mg/dL were diagnosed as having iron deficiency anemia. Oral elemental iron treatment was started at 4 mg/kg per dose in all cases.

Echocardiography and electrocardiography were evaluated by the cardiology department for the exclusion of cardiac pathologies. The QT interval was calculated in terms of long QT syndrome using Bazett’s formula. Electroencephalographic evaluation was performed by our hospital’s pediatric neurology specialists. EEG findings were examined within three groups, 1- normal, 2- slow waves and background rhythm abnormality, and 3- epileptic anomalies.

Patients were called for follow-up appointments 3 months after the initiation of treatment and their hematological parameters were evaluated again. Thirty-eight of the patients were excluded from the study due to failure to complete tests or attend follow-ups. 

### 2.1. Statistical analysis

SPSS 24.0 (IBM Corp., Armonk, NY, USA) and PAST 3 (https://folk.uio.no/ohammer/past/) software were used for variable analysis. Normality of distribution was evaluated using the Shapiro–Wilk test for single-variable data and Mardia’s test (Dornik and Hansen omnibus) for multivariable data, while variance homogeneity was assessed with the Levene test. The Mann–Whitney U test and Monte Carlo results were used together in the comparison of quantitative data from two independent groups. The Kruskal–Wallis H test and Monte Carlo results were used together in the comparison of quantitative data from more than two independent groups. The Wilcoxon signed ranks test and Monte Carlo results were used in the comparison of two repeated measurements from dependent quantitative variables. The marginal homogeneity test was used with the Monte Carlo simulation technique. Quantitative variables were expressed as mean ± standard deviation (SD), median ± interquartile range (IQR), and median range (maximum–minimum), while categorical variables were expressed as n (%). Variables were analyzed at a 95% confidence level, and P-values less than 0.05 were regarded as statistically significant.

## 3. Results

Cyanotic breath-holding spells were diagnosed in 85.3% (n = 266) of patients, pallid spells in 5.1% (n = 16), and mixed-type spells in 9.6% (n = 30). These patients’ laboratory findings and demographic data were compared with those of 100 patients undergoing simple febrile convulsions (Table 1). 

**Table 1:** Demographic data of breath-holding spell and control groups.

		Breath-holding spells, n (%)	Control, n (%)	P-value
Age	<24 months >24 months	202 (64.7) 110 (35.3)	70 (70) 30 (70)	0.480
Sex	FemaleMale	132 (42.3)180 (57.7)	42 (42)58 (58)	0.1
Anemia	Absent Present	55 (17.6) 257 (82.4)	71 (71) 29 (29)	<0.001
Anemia after iron treatment	Absent Present	255 (81.8) 57 (18.2)	- -	
Developmental delay	Absent Present	306 (98.1) 6 (1.9)	100 (100) 0 (0)	0.343
Perinatal problems	Absent Present	303 (97.1) 9 (2.9)	96 (4) 6 (6)	0.526
Consanguinity	Absent Present	159 (51) 153 (49)	57 (57) 43 (43)	0.303
Epilepsy in first-degree relatives	Absent Present	288 (92.3) 24 (7.7)	89 (89) 11 (11)	0.306

Onset of spells occurred below 24 months in the great majority of cases (64.7% [n = 202]). This group’s mean Hb concentration was 9.8 mg/dL (P = 0.045). Breath-holding spells beginning to be observed after the age of 6 months suggested an association with inadequate iron support in the 4th month, when childhood physiological anemia is seen. 

Patient and control group Hb and MCV values are shown in Table 2. The mean Hb value in the patient group was 10.1 mg/dL. When subjects were considered in terms of appropriateness for age, Hb values were below –2 SDs in 82.4% (n = 257) of patients. Patients’ mean MCV value was 73 fL. Patient group Hb and MCV values were significantly lower compared with those of the control group (P < 0.001). In the patient group 55 patients had no anemia. Twenty-eight patients had Hb levels below 7 mg/dL and 68 patients had MCV below 70 fL (Table 3).

**Table 2:** Demographic data and laboratory values of breath-holding spell group.

		n (%)	Mean ±SD	Median (max/min)
Ferritin		312	20.38±9.60	20 (52/4)
QTc		312	409.64±29.37	421 (449/350)
Month of onset of spells		312	9.44±3.58	9 (20/2)
Pretreatment spell burden		312	20.56±24.73	10 (100/1)
Posttreatment spell burden		312	3.97±1.98	2 (30/0)
Difference of spell burden betweenpre- and posttreatment		312	16.58±3.58	10 (99/-8)
Pretreatment spell duration (s)		312	28.56±7.93	15 (120/5)
Posttreatment spell duration (s)		312	5.80±1.79	5 (45/0)
Difference of spell duration betweenpre- and posttreatment (s)		312	-22.76±8.11	–10 (10/–120)
Type of breath holding spell	Cyanotic Pallid Mixed	266(85.3) 16(5.1) 30(9.6)		
Type of breath holding spell-2	Complicated Simple	77 (24.7) 235(75.3)		
Response to treatment	Complete response >%50 decrease <%50 decrease No response	147(47.1) 122(39.1) 23(7.4) 20(6.4)		
BHS in first-degree relatives	Present Absent	44(14.1) 268(85.9)		
History of seizure	Present Absent	10 (3.2) 302(96.8)		
Electroencephalography	Normal Slow waves Epileptic activity	306(98.2) 4(1.2) 2(0.6)		
Echocardiography	Normal PFO VSD ASD	291(93.3) 15(4.8) 3(1) 3(1)		

SD: Standard deviation, Max: maximum Min: minimum, MCV: mean corpuscular volume, BHS: breath holding spell, PFO: patent foramen ovale, PDA: patent ductus arteriosus, VSD: ventricular septal defect, ASD: atrial septal defect.

**Table 3:** Comparison of hemoglobin, mean corpuscular volume, ferritin, QTc, and anemia and iron deficiency presence in terms of breath-holding spell type, spell burden, and duration.

	Breath-holdingspell type (n=312)		P-value	Spell burden(per month)		P-value	Spell duration (s)		P-value
	Complicated	Simple		>10	<10		>30	<30	
	Median ± IQR			Median ± IQR			Median ± IQR		
Hemoglobin(mg/dL)	10.2 ± 1.7	10.1 ± 1	0.833	9.2 ± 1	11.4 ± 1	0.042	9.1 ± 1	11 ± 1.2	0.032
Mean corpuscular volume (fL)	72 ± 8	73 ± 6	0.286	65 ± 3	74 ± 4	0.038	64 ± 4	75 ± 4	0.040
Ferritin (g/L)	20 ± 11	20 ± 11	0.517	11 ± 2	31 ± 4	0.002	10.2 ± 1	33 ± 2	0.001
QTc (ms)	421 ± 50	389 ± 39	0.003	412 ± 32	398 ± 42	0.567	405 ± 23	412 ± 12	0.65
	Breath-holding spell type		P-value	Spell burden (per month)		P-value	Spell duration (s)		P-value
	Complicated	Simple		>10	<10		>30	<30	
Anemia + (n = 257)	77	180	0.076	92	135	0.082	35	222	0.065
Iron deficiency + (n = 287)	77	210	0.089	149	138	0.380	192	95	0.780

Seventy-seven patients’ spells were longer than 30 s, 50 of them were described as complicated type, and this group’s median Hb value was 9.1 mg/dL. Median Hb value of the patients who had spells less than 30 s was 11 mg/dL (P = 0.032) (Table 3). Sleep EEG was performed for all patients and results were normal in 98.2% (n = 306) of patients, while slow background rhythm was determined in 1.2% (n = 4). Epileptic activity was observed in only 2 (0.6%) patients. Both epileptic activity and breath-holding spells were observed in one of these, and the patient was started on oral iron and antiepileptic therapy. No pathology was determined in these patients during cranial magnetic resonance imaging (MRI) performed to determine epileptic focus. The patients’ breath-holding spells resolved with iron therapy. The other patient is still being monitored without antiepileptic therapy.

ECG and echocardiography were performed for all patients. QTc values were not above normal in any patients. The patients’ mean QTc value was 409.64 ms. Significant prolongation was determined in QTc values in subjects with complicated breath-holding spells compared to those with the simple type (P = 0.003) (Table 3). Pathology was determined in echocardiography in 21 (6.8%) patients, consisting of patent foramen ovale in 15 (4.8%), secundum atrial septal defect in 3 (1%), and ventricular septal defect in 3 (1%). The patients with pathology identified in echocardiography continue to be monitored by the cardiology and pediatric neurology departments. 

All the patients included in the study received 4 mg/kg oral iron therapy daily. However, since 20 patients exhibited no response to iron therapy, piracetam therapy was added at the end of the third month. When the patients’ spell burden and duration were compared before and after treatment, it was observed that pretreatment spell burden decreased from 10 spells/month to 2 and the duration of spells decreased from 15 s to 5 s (Tables 4 and 5). Statistically significant decreases were observed after treatment in attack numbers and durations (P < 0.001 for both). Patients in the breath-holding spell group were compared among those with and without anemia. There was no significant difference between the two groups’ spell burden after iron treatment (P = 0.691). Spell burden decreased equally in both groups. 

**Table 4:** Alteration of spell count and duration before and after iron treatment.

Breath-holdingspell (n=312)		Pretreatment	Posttreatment	P-value
		Median ± IQR		
Spell burden		10 ± 15	2 ± 6	<0.001
Spell duration (s)		15 ± 35	5 ± 10	<0.001
		n (%)		
Spell burden				
	>30 spells/month	27 (8.7)	0 (0)	<0.001
	10–30 spells/month	122 (39.1)	51 (16.3)	
	<10 spells/month	163 (52.2)	261 (83.7)	

**Table 5:** Alteration of hematological parameters, spell burden, and duration in breath-holding spell group before and after iron treatment in terms of anemia.

	Breath-holding spell group		
	Anemia (-) n = 55	Anemia (+) n = 257	P-value
	Mean (±SD)	Mean (±SD)	
Hemoglobin (mg/dL)	12.7 (±4)	9.1(±2)	0.03
<7	0	27	
>7	55	230	
Hematocrit	38 (±4)	29 (±5)	<0.001
Mean corpuscular volume (fL)	81 (±12)	69 (±14)	0.045
<70	0	68	
>70	55	189	
Ferritin (g/L)	22 (±6)	9 (±2)	<0.001
Iron (μg/dL)	38 (±14)	27 (±9)	0.098
Total iron-binding capacity	360 (±50)	389 (±59)	0.567
	Median ± IQR	Median ± IQR	
QTc (ms)	425 ± 54	421 ± 44	0.060
Onset of spells (month)	9 ± 6	9 ± 5	0.744
Pretreatment spell burden	2 ± 5	2 ± 10	<0.001
Posttreatment spell burden	2 ± 10	2 ± 5	0.908
Difference of pre- and posttreatment spell burden	10 ± 20	10 ± 16	0.691
Pretreatment spell duration (s)	5 ± 10	10 ± 16	<0.001
Posttreatment spell duration (s)	2 ± 5	5 ± 10	0.038
Difference of pre- and posttreatment spell duration (s)	–9.5 ± 15	–11.3 ± 35	0.049

Fifty-seven (18.2) patients still had anemia after 3 months with iron supplementation. 

## 4. Discussion

This study investigated the laboratory findings of patients diagnosed with breath-holding spells and short-term response to iron supplementation. Breath-holding spells are a nonepileptic paroxysmal phenomenon frequently seen in childhood, particularly at 6–48 months. Although the condition is a source of anxiety for families, it is frequently described as benign. The earliest age at onset was reported in a 3-day-old newborn with a family history (4). The youngest patient in our study was aged 2 months. No statistically significant difference was determined in terms of sex in our study. Mean age at presentation was 10.3 months, and mean age at onset of symptoms was 9.4 months. In agreement with our findings, the great majority of patients in previous studies have been under 1 year of age. The fact that breath-holding spells frequently commence after 6 months suggests a possible association with insufficient iron support at 4 months, when childhood physiological anemia is seen, and problems regarding the transition to supplementary foods. 

Epilepsy was present in first-degree relatives in 7.7% (n = 24) of patients. This figure was markedly lower than in other studies (8). This suggests that breath-holding seizures may have been misdiagnosed as epilepsy. Studies have stated that breath-holding spells are inherited in an autosomal dominant manner (8–10). In our study, breath-holding spells were present in 14.1% (n = 44) of first-degree relatives of our patients.

The proportion of cyanotic breath-holding spells in previous studies ranges between 58% and 86.1%, and these seizures were also predominant in our study, at 85.3% (n = 266) (2,11,12). This predominance may be due to cyanosis causing greater alarm among families and leading them to feel that ‘the child is about to die’, thus resulting in higher numbers of hospital presentations. Spell burden ranges from ‘a few times every day’ to ‘one attack in a lifetime’. Spell durations in the literature range between 40 and 60 s, the mean duration in our study being 28.56 s. Spell burden and durations both decreased with oral iron therapy.

Iron deficiency has long been known to represent a risk factor for breath-holding spells (13). Iron deficiency anemia was determined in 82.4% (n = 257) of our patients. Lower prevalences of 47.9%–73% have been reported in other studies (14,15). The higher rate in our study may derive from our hospital being in a province of Turkey with a low socioeconomic level. Anemia is thought to represent the principal cause of decreased cerebral blood flow. Iron is also known to be involved in catecholamine metabolism, and changes occur in neurotransmission in the central nervous system in the event of iron deficiency (16). Clinical and laboratory findings have been reported to be potentially associated with cerebral erythropoietin, nitric oxide, and interleukin-1 levels (16,17). Additionally, animal experiments showed changes in cerebral dopamine metabolism in individuals with iron deficiency (18,19). EEGs of children with iron deficiency had slower activity than those of the control group, which could be associated with central nervous system dysfunction (20). Besides this, febrile convulsions were more frequent in patients with iron deficiency (21). In light of all this information, it can be beneficial to administer iron therapy in reducing seizure burden regardless of anemia in breath holding spells.

Lombrosso and Lerman also stated that iron deficiency is not the sole cause of breath-holding spells, and that iron deficiency also exacerbates the underlying pathology (22). In our study, all patients, regardless of anemia, were therefore started on 4 mg/kg oral elemental iron supplement daily, based on the information in the previous literature. After 3 months, seizure frequency had decreased by 50% or more in 86.2% of patients. Previous studies have reported response rates to iron therapy of 66.6%–96%, and our finding is in agreement with these (8,14,15). Interestingly, comparison of responses to iron therapy among patients with and without anemia revealed that both spell burden and spell duration decreased in both groups, although these results were not statistically significant. This shows that iron therapy can be effective in breath-holding spells even if the patient is not anemic. In their controlled study of 67 patients with breath-holding spells, Daoud et al. reported that subjects with iron deficiency benefitted more from iron therapy then those without (23). To the best of our knowledge, no previous studies have compared breath-holding spells in patients with and without anemia.

Many breath-holding spells may be misdiagnosed as epileptic seizures, due to both a lack of knowledge and the fact that the attack is not observed by a health worker, and patients may be incorrectly started on antiepileptic therapy. The two conditions can be easily differentiated by means of history. EEG is frequently used to distinguish epileptic seizures from breath-holding spells. EEG was performed for our entire patient group, and epileptic activity was determined in only 0.6% (n = 2). No pathology was determined in MRI preformed due to epileptic focus being observed in these two patients. Comorbid epileptic seizures and breath-holding spells were determined in one case, and this patient was started on phenobarbital in addition to iron therapy. Although pathology was determined in EEG in the other case, that patient was not started on antiepileptic therapy since the history was compatible with breath-holding spells. The patients’ spells resolved entirely with iron therapy. EEG is impracticable in the diagnosis of breath-holding spells, since it is difficult to perform with children and requires sedation, and also since it is not more useful than history-taking.

Arrhythmias and particularly long QT syndrome are useful in the differential diagnosis of breath-holding spells. Autonomic nervous system dysfunction leading to QT prolongation at the time of attack has been implicated, particularly in pallid breath-holding spells (24). This QT prolongation can lead to fatal arrhythmias and sudden cardiac arrest in cases with familial long QT syndrome. ECG and echocardiography were therefore performed in all cases. No long QT syndrome was determined in any patient. Although the absence of rhythm abnormality in any out of 312 patients might at first sight appear insignificant, preventing the sudden death of a child with a simple test is of great importance. The presence of severe, life-threatening arrhythmias, such as Wolf–Parkinson–White and long QT syndrome, has also been shown in previous studies, as in the nonarrhythmia group (25). In a cardiac repolarization study performed with “event recorders” in breath-holding spells, autonomic dysregulation was observed in patients compared to the control group. It was argued that iron deficiency may have had an effect on this autonomic change (26). It was reported that patients with prolonged and frequent spells had longer QT and T-wave dispersion than the control group (27). Akalın et al. compared 43 patients with breath-holding spells and 25 healthy children and determined statistically significantly longer QT and QTc dispersion in the patient group than in the controls. This also shows an autonomic effect and arrhythmogenic potential in breath-holding spells (28). Pathology was determined in echocardiography in 6.8% (n = 21) of patients, and these were also not pathologies that could be linked to breath-holding spells. 

As explained above, EEG, ECG, and echocardiography are not diagnostic in breath-holding spells. However, a good history is diagnostic. Asking the mother questions such as ‘Do the attacks always occur following an emotional stimulus or trauma?’ and ‘Do they occur during sleep?’ is to a large extent diagnostic of breath-holding spells. As also described, breath-holding spells frequently occur after an emotional and traumatic event, but never in sleep. History is therefore the gold standard. Studies have shown that piracetam therapy can be useful if no benefit is achieved with oral iron therapy in the first instance (29). Giving the family appropriate information is also of great importance in addition to medical treatment.

In conclusion, breath-holding spells can be diagnosed with an accurate history. EEG and echocardiography are of no diagnostic value. ECG, which is cost-effective and can be easily performed in any center, is important in terms of preventing rare fatal complications. Iron therapy should be given in all breath-holding spells, regardless of anemia, because it is much more related with iron stocks than hemoglobin.
